# Ytterbium-doped fibre femtosecond laser offers robust operation with deep and precise microsurgery of *C. elegans* neurons

**DOI:** 10.1038/s41598-020-61479-0

**Published:** 2020-03-11

**Authors:** M. B. Harreguy, V. Marfil, N. W. F. Grooms, C. V. Gabel, S. H. Chung, G. Haspel

**Affiliations:** 1 New Jersey Institute of Technology and Rutgers University, Federated Department of Biological Sciences and New Jersey Institute of Technology, Institute of Brain Research and Neuroscience, 100 Summit St, Newark, NJ 07102 USA; 2 Northeastern University, Department of Bioengineering, 360 Huntington Avenue, Boston, MA 02115 USA; 3 Department of Physiology and Biophysics and Department of Pharmacology and Experimental Therapeutics, Boston University School of Medicine and Boston University Photonics Center, 700 Albany St, Boston, Massachusetts 02215 USA

**Keywords:** Microscopy, Neuroscience

## Abstract

Laser microsurgery is a powerful tool for neurobiology, used to ablate cells and sever neurites *in-vivo*. We compare a relatively new laser source to two well-established designs. Rare-earth-doped mode-locked fibre lasers that produce high power pulses recently gained popularity for industrial uses. Such systems are manufactured to high standards of robustness and low maintenance requirements typical of solid-state lasers. We demonstrate that an Ytterbium-doped fibre femtosecond laser is comparable in precision to a Ti:Sapphire femtosecond laser (1–2 micrometres), but with added operational reliability. Due to the lower pulse energy required to ablate, it is more precise than a solid-state nanosecond laser. Due to reduced scattering of near infrared light, it can lesion deeper (more than 100 micrometres) in tissue. These advantages are not specific to the model system ablated for our demonstration, namely neurites in the nematode *C. elegans*, but are applicable to other systems and transparent tissue where a precise micron-resolution dissection is required.

## Introduction

A focused pulse of laser light can induce accurately localized sub-cellular damage to approach diverse questions in biology. In the field of neuroscience, laser ablation can kill cells (*i.e*., neurons and glia) by aiming at the nucleus, or sever neurites (*i.e*., axotomy and dendrotomy), allowing the study of neuronal function and regeneration *in-vivo*. Laser microsurgery platforms often use pulsed lasers at nanosecond or femtosecond pulse width. The pulses’ high strength electric field ionizes molecules to create a bubble of plasma that vaporizes water and tissue to generate damage^1,2^. The absorption process at the focus is non-linear and depends strongly on the pulse intensity, which scales with the pulse energy divided by pulse duration^3^. Hence, the ten-thousand-fold shorter femtosecond laser pulse requires lower energy for plasma formation and ablation. Pulse energies used for femtosecond pulse ablation are typically tens of nanojoules (nJ), compared to tens of millijoules (mJ) for nanosecond pulse ablations. Excess deposited energy diffuses away from the focal point. Accordingly, longer pulses generate more severe damage beyond the region of laser energy deposition compared to shorter pulses^4^.

Laser microsurgery was pioneered and has been an important tool to study the development and neurobiology of *Caenorhabditis elegans* since 1980 when Sulston and White used it to study cell-cell interaction in post embryonic stages^5^, and when Chalfie and colleagues ablated neurons to test their necessity for touch sensitivity^6^. Microsurgery of neurites (termed axotomy) was also pioneered in *C. elegans*^7^ where a laser-pumped titanium-sapphire laser was used to cut commissure neurites of motoneurons. These Ti:Sapphire lasers are typically configured to produce near-infrared (NIR) pulses with energies up to 50 nJ, a centre wavelength of approximately 800 nm, pulse duration of 100–200 femtoseconds, and repetition rates of 80 MHz. Ablation at 80 MHz does not allow complete dissipation of pulse energy^8^ and in many cases an external electro-optic pulse picking device is added to reduce the repetition rate and ablate at 1 to 10 kHz. The longer intervals between pulses at these lower repetition rates allow the deposited energy to dissipate completely^8–10^, improving the surgical resolution. Nanosecond lasers were adapted for axotomy, offering lower cost, and more robust design^6,11^, but with higher energy per pulse. They are typically diode laser-pumped or Nitrogen laser-pumped dye laser typically configured to produce green (532 nm), blue (450 and 488 nm), or ultra violet (337 nm) light. These lasers have pulse energies up to several mJ, pulse durations of several nanoseconds, and repetition rates up to 10 kHz.

Axotomy using nanosecond and femtosecond pulses at high or low repetition rate varies in the extent of damage to surrounding tissues and in the size of the gap induced in a severed axon, but axon regeneration seems to occur at comparable extents and rates after axotomy^12^. At low repetition rate (1 kHz), the size of damage to tissue and neurite regeneration rates appear to be mostly affected by pulse energy^13^. Laser energies near the damage threshold improve surgical resolution. For any energy level there is a minimal number of pulses that will initiate ablation but adding pulses does not substantially increase the region damaged^3,13^.

Nanosecond lasers advantages are their relatively low cost, compact size, low maintenance and lower safety requirements. Femtosecond lasers dissect with higher resolution and less damage to surrounding tissues. The choice of laser system is usually motivated by application and involve a compromise, considering precision, cost, and operational reliability. Neurites spaced further apart require less surgical resolution while bundled neurites often require submicron resolution^10^. Here we compare a new type of laser, an Ytterbium-doped fibre femtosecond-pulse laser, with the two laser types most commonly used for laser axotomy in *C. elegans*: a femtosecond Ti:Sapphire and a nanosecond diode-pumped passively Q-switched solid state laser.

## Results

### Ytterbium-doped fibre femtosecond laser

**Figure 1:**
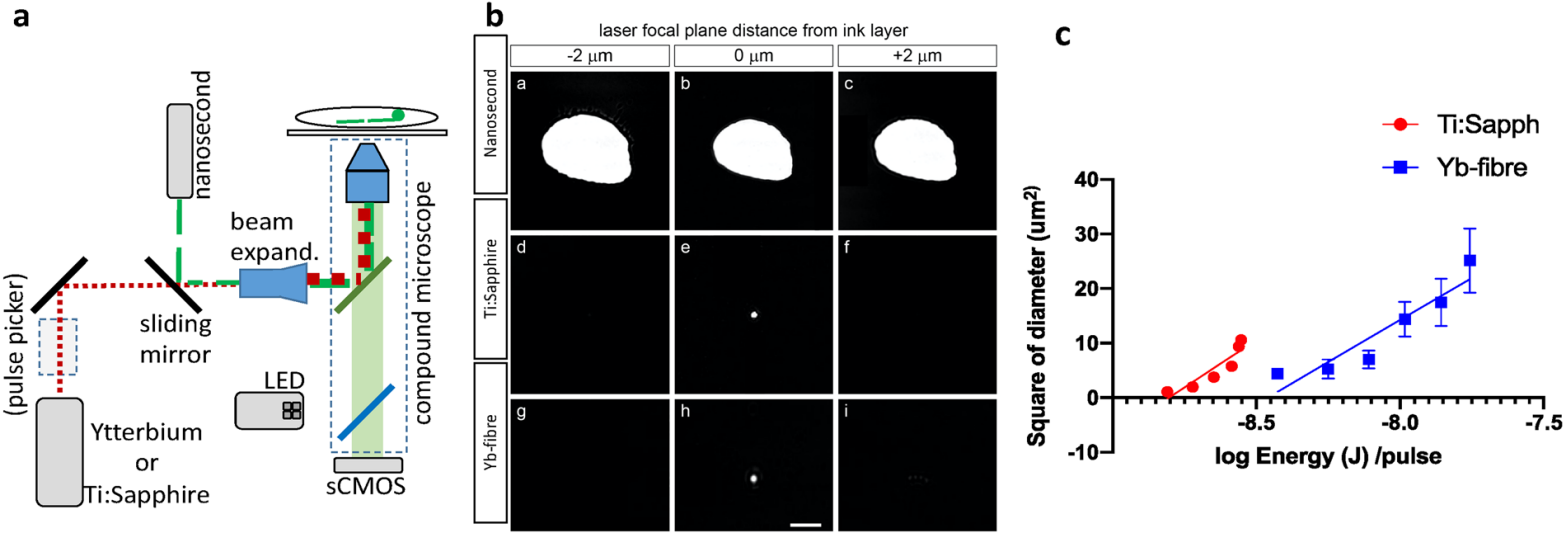
Yb-doped fibre femtosecond laser integrated in epifluorescence microscope produces a small damage spot, comparable to Ti:Sapphire laser. (**a**) Lasers are integrated to an epifluorescence compound microscope through a beam-splitter or dichroic mirror. Note that some lasers require an external pulse picking device. (**b**) Damage spot on a thin layer of black ink is larger when induced with a nanosecond pulse laser (top) than with either of the femtosecond lasers (middle Ti:Sapphire; bottom Yb-fibre). Damage spots are not reduced in size two micrometres above or below the image focal plane for the nanosecond pulse laser, while undetected for the femtosecond pulse lasers. Scale bar = 5 μm. (**c**) Square diameter of damage plotted against log scale of the energy per pulse used to estimate theoretical minimum beam size (square root of slope) and the threshold energy (x-axis intercept) for Ti:Sapphire (red) and Yb-fibre (blue).

Rare-earth doped mode-locked fibre laser produces high power pulsed radiation by amplifying seed source radiation through a thin coiled fibre. Specifically Yb_2_O_3_-doped fibre lasers emit pulses that are one hundred to hundreds of femtoseconds wide, with wavelengths 1000–1100 nm, at average power of milliwatts to tens of watts^14^. The basic design is similar to Ti:Sapphire lasers that are very commonly used for multiphoton imaging and axotomy^15,16^. One advantage of Ti:Sapphire laser over Yb-fibre is that the wavelength is tunable, but this advantage is not crucial for ablation applications such as ours. The advantages of Yb-fibre are higher possible power, lower maintenance, smaller footprint, and air cooling. The specific system used here (BlueCut, Menlo Systems GmbH, Germany) includes an internal pulse picker which simplifies set up and lowers the overall cost compared to Ti:Sapphire systems that require an external pulse picker. Our Yb-fibre system can ablate with user-defined repetition rate of single shot to 50 MHz and pulse energies of nJ to μJ. As further described in the Methods, in the Yb-fibre ablation setup the laser beam is sent through a beam expander into a microscope objective that focuses the pulses into a sample. Brightfield and fluorescent light is captured by a sCMOS camera for visualization and targeting (Fig. 1a).

### Lesion size

We evaluated lesion size by focusing the laser beams through a coverslip onto a layer of black ink (Fig. 1b). We adjusted the laser to the lowest possible power setting that induced axotomy. Yb-fibre laser produced a slightly smaller damage at the focal plane (Fig. 1e; 1.34 ± 0.25 μm; n = 15; p = 0.04) than Ti:Sapphire (Fig. 1h; 1.66 ± 0.36 μm; n = 16); neither generated significant damage 2 μm above or below the focal plane (Fig. 1b.d,f,g,i). The nanosecond-pulse laser produced a much larger lesion than either femtosecond laser (p < 0.0001) not only at the focal plane (12.5 ± 0.5 μm) but also 2 μm above or below (13.0 ± 0.4 μm; 14.0 ± 0.4 μm). Even though smaller lesion sizes were reported for the nanosecond laser, that study utilized a very low energy setting (10 pulses at 100 Hz) only meant for alignment and not for axotomy^11^.

To calculate the minimum theoretical beam size diameter as well as the threshold energy we used the extrapolating method described by Liu *et al*.^17^. As shown in Fig. 1c, we plotted square diameter of damage area against the log of the pulse energy and fit data to a line. We calculated the theoretical minimum beam size as the square root of the slope of the line. We calculated the threshold energy as the x-axis intercept of the same line (Fig. 1c). The calculated minimum diameter was 5.9 ± 2.1 µm for Ti:Sapphire and 5.6 ± 2.2 µm for Yb-fibre; threshold energy was 1.57 ± 0.54 nJ and 3.4 ± 0.8 nJ respectively.

We estimated the peak intensities by modelling the pulse intensity, I(x, y, t), as Gaussians in the transverse directions (x,y) and in time (t)^18^. The Gaussians are defined by full width at half maximum (FWHM) in each direction. The FWHM in the transverse directions are the minimum diameter, d, calculated from the fit in Fig. 1c. The FWHM in the temporal direction is the pulse duration, τ. Integrating I(x, y, t) over all x, y, and t, we find that the pulse energy E = I_0_ d^2^ τ (π/ln(16))^3/2^, where I_0_ is the peak intensity. By using the threshold energy calculated from Fig. 1c and assuming pulses without optical stretching (100 fs for Ti:Sapphire, and 400 fs for Yb-doped laser), we obtain I_0_ = 3.7 × 10^10^ W/cm^2^ for Ti:Sapphire and 2.2 × 10^10^ W/cm^2^ for Yb-doped fibre laser. Because some linear absorption of laser light by the black ink may occur, the threshold energy is likely underestimated and the minimum diameter is likely overestimated, so that I_0_ is higher for cells.

### Qualitative accuracy test by dendritic bundle ablation in *C. elegans*

**Figure 2:**
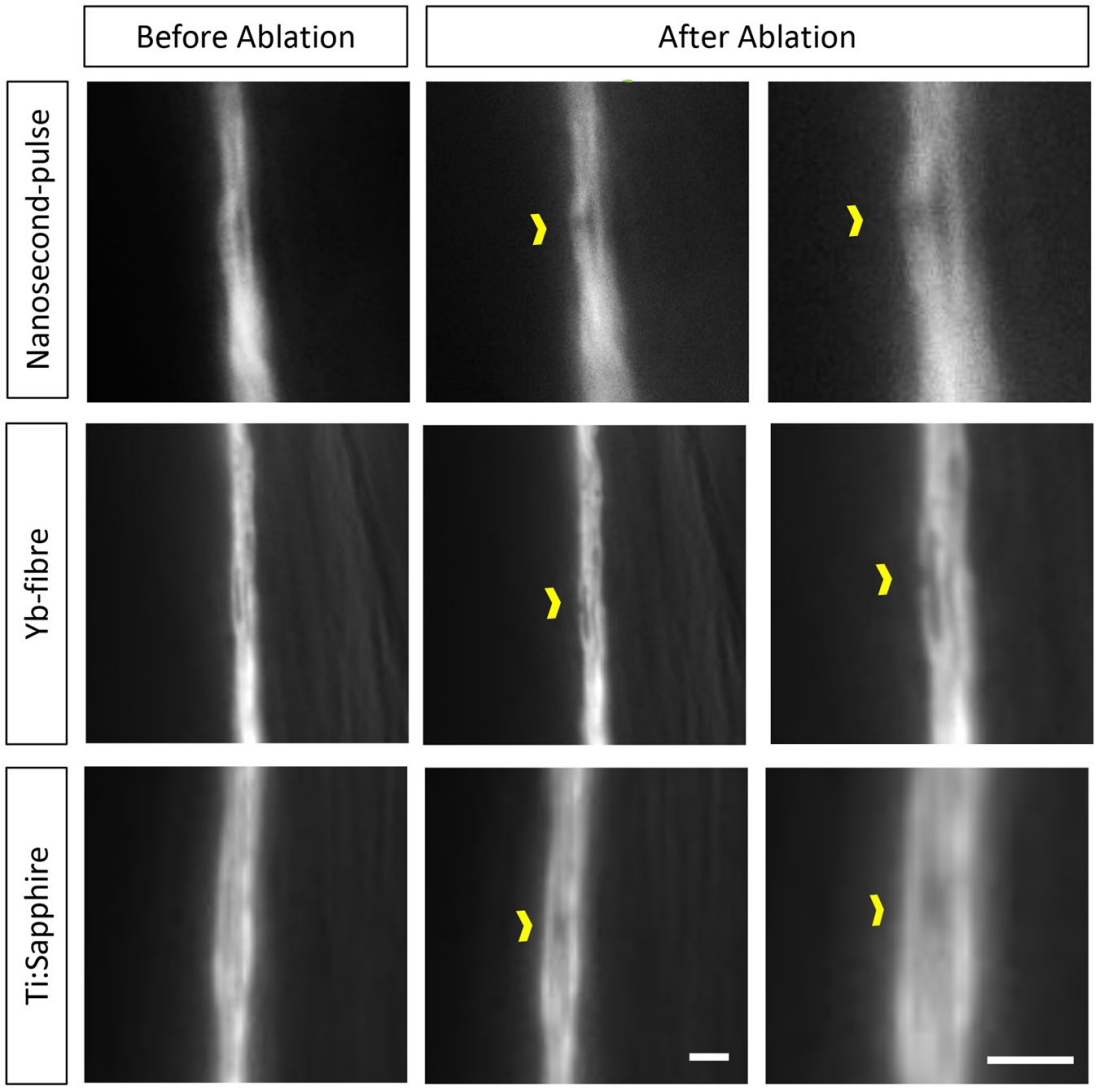
A single *C. elegans* dendrite can be injured without damage to neighbouring dendrites. A single dendrite located in a sensory bundle in the nose of the animals was successfully ablated (yellow chevron) using the Ti:Sapphire and the Yb-fibre lasers without collateral damage to adjacent dendrites. The nanosecond-pulse laser injured more than one dendrite. Scale bar = 2 μm.

A Ti:Sapphire laser system can selectively ablate one sensory dendrite in a tight bundle of twelve dendrites^10^. These dendrites are sensory organs of the twelve amphid neurons located in the nose of *C. elegans*. We replicated this treatment using both the nanosecond pulse laser and the Yb-fibre laser. The Yb-fibre femtosecond laser, similar to the Ti:Sapphire, is capable of ablating only one neurite in the bundle with no damage to surrounding tissue. However, the nanosecond-pulse laser produces a larger injury affecting surrounding tissue and therefore damages more than one dendrite (Fig. 2). The damage observed for the nanosecond-pulse laser is more localized than what we previously described in Fig. 1b. This could be due to the fact that the higher energy might be causing thermal damage to the ink while not vaporizing the tissue. Additionally, the three-dimensional geometry and the ability of the energy to penetrate through the tissue also need to be considered.

### Quantitative regeneration assessment in* C. elegans* motoneurons

**Figure 3:**
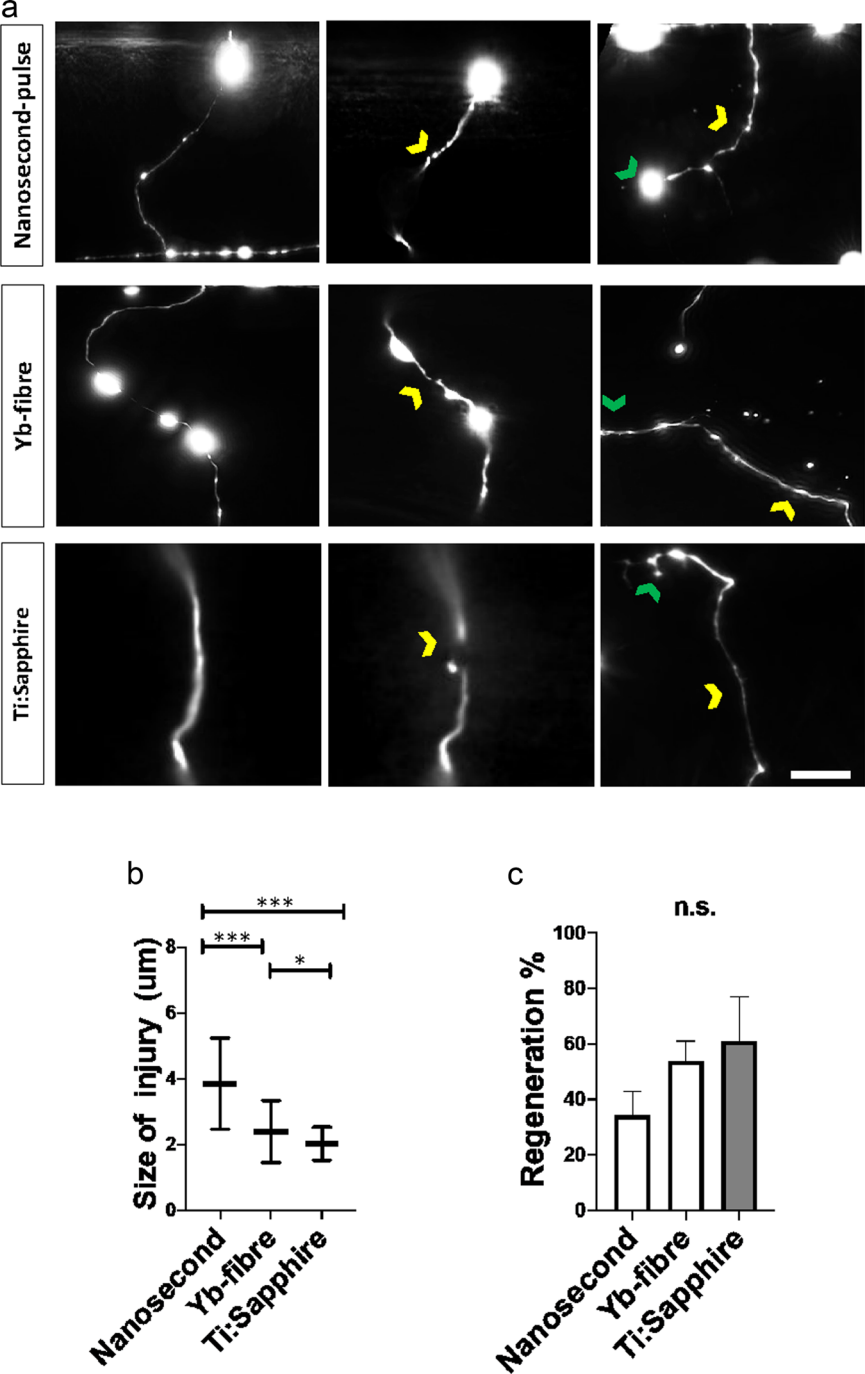
Nanosecond laser induces larger gap at site of injury but injured neurons regenerate at similar rate. (**a**) *C. elegans* motoneuron axotomy. Commissures of D motoneurons in immobilized animals were axotomized, animals were recovered and axon found again next day to asses regeneration. Images taken before, 1 second after and 24 hours after injury. Sites of axotomy (yellow chevron) and regenerating branch (green chevron) are indicated. (**b**) Mean gap between injured tips was larger after injury with nanosecond laser. (**c**) Percentage of neurons that regenerated at 24 h was the same for the three types of lasers. Images in A for before and at 24 h are maximal projections of Z stacks while the images at 1 s are each a single frame, leaving some parts of the neuron out of the imaging plane. *p < 0.05 ***p < 0.001. Scale bar. = 5 μm.

Finally, we assessed how the different laser setups might impact neuronal regeneration in *C. elegans* (Fig. 3). We aimed the three laser systems at commissures of D-type motoneurons which extend from the ventral to dorsal side of the animal (Fig. 3a). The size of the axotomy-gap (Fig. 3b) between severed ends produced by the nanosecond laser after initial retraction (3.8 ± 1.4 μm) was significantly larger than the gap produced by Yb-fibre laser (2.4 ± 0.8 μm, p = 0.02) or Ti:Sapphire laser (2.03 ± 0.49 µm, p < 0.0001). However, similar proportion of neurites regenerated after injury (Fig. 3c; p = 0.12) with the Yb-fibre laser (27 of 50 neurons in 11 animals), nanosecond laser (11 of 32 neurons in 11 animals), or Ti:Sapphire laser (16 of 26 neurons in 9 animals).

## Discussion

Yb-doped fibre lasers possess several advantages over more commonly used nanosecond pulse lasers and similar capabilities to Ti:Sapphire lasers. Femtosecond fibre laser systems are designed for the high standards of industrial cutting and welding. Compared to a Ti:Sapphire system, Ytterbium-doped fibre laser requires much less frequent alignment and optimization and less maintenance overall. It is our experience that a Ti:Sapphire system requires daily alignment to maintain its operation, but a Yb-doped system turned off for weeks could cut axons as soon as it was turned back on. Comparable turnkey laser systems, such as the Spectra-Physics Spirit, are commercially available. However, we found that the BlueCut Yb-fibre laser was the most suitable at the time we initiated this study. The most notable reasons include the integrated pulse picking, the simple incorporation of this unit to an existing microscope, air rather than liquid cooling and the relatively lower cost (including the external pulse-picker required for other systems). The Yb-fibre laser used here offers integrated power adjustment and pulse picking, controlled by a dedicated software that can pick single pulses. In our experience, we never use the full laser power and a unit with about 20% of existing power would have been preferable, for safety and cost reasons.

The ability to produce injury depends on pulse energy and duration. The injury producing plasma is induced by a multiphoton absorption that requires a minimal intensity threshold which is only reached at the focal plane. In the case of neuronal injury with a 1.4 NA objective and a pulse duration of 100 fs, this threshold occurs at an intensity of 6.5 × 10^12^ W/cm^2^ or an energy of 4 nJ per pulse^1^. In this study, Yb-fibre and Ti:Sapphire lasers produced comparable pulse width (<400 fs), while the nanosecond-pulse laser produced four orders of magnitude longer pulse (<1.3 ns). Hence, the former induces axotomy with lower energy levels and therefore less collateral damage as longer pulses require more energy to reach the threshold intensity. Yet, regeneration rates were comparable among all three systems.

Further, the Yb-fibre laser system enables the lesion of neuronal processes located on the side of an adult *C. elegans* farther from the objective (n > 20 animals). The mounting and diameter of an adult animal requires optical penetration of 80–100 μm deep, compared to 30–40 microns for nanosecond laser systems^11^. The most probable reason is that near infrared photons penetrate deeper and are less scattered by biological tissue. Reduced scattering also lowers the required energy levels.

Here we have described a new laser axotomy system that provides the same high precision capabilities of widely-used Ti:Sapphire lasers but with less maintenance and higher robustness. This capability is not limited to neurons in *C. elegans* and might be useful to address experimental questions in transparent tissue that require an accurate and localized micrometre-sized lesion up to 100 μm deep with no collateral damage.

## Methods

### Laser platforms

The Yb-fibre system which generates ~400 fs pulses in the infrared (1030 nm) (BlueCut, Menlo Systems GmbH, Germany) includes an integrated pulse picking unit composed of two acousto-optic modulators (AOMs). A fixed AOM reduces repetition rate from 50 MHz to 1 MHz, a second variable AOM reduces it further as low as 1 kHz. We use the BlueCut Control software provided by Menlo Systems with the laser. The software allows user control of the repetition rate (typically, we use 1 kHz) and external gating of the variable AOM by a transistor-transistor logic (TTL) signal. We use a function generator (model 33210 A, Keysight Technologies) to provide the TTL pulse at various lengths (typically 100 ms for 100 pulses at 1 kHz). Reliable picking of single laser pulses can be accomplished through synchronization of the two AOMs in the pulse picker unit. Alternatively, we pick a single laser pulse with a 1-ms TTL signal, accepting occasional fails that are easy to visually recognize. Users control laser power by choosing a level of 1 to 99 arbitrary units that are scaled by the software. Typically, we use 10–20 units for ablations. We measured 3–65 nJ/pulse at the image plane (PM100D Power and Energy Meter Console with S170C Microscope Slide Power Sensor Thorlabs GmbH) when changing the arbitrary units scale from 4 to 35 (of 99).The beam is directed through a beam expander (10X Achromatic Galilean Beam Expander, AR Coated: 650–1050 nm, Thorlabs, USA) and dichroic mirror (750 nm long-pass, Thorlabs, USA).

The diode pumped passively Q-switched solid state system (1Q532–3), Crylas Laser Systems, USA^11^, was integrated via a flip-mounted mirror to the same optical path and microscope objective as the Yb-fibre laser. The only components that had to be replaced were the beam expander (10X Achromatic Galilean Beam Expander, AR Coated: 400–650 nm, Thorlabs, USA) and 1:1 beam splitter (Thorlabs, USA) to accommodate the shorter wavelength (532 nm).

The Ti:Sapphire femtosecond laser (Mantis Pulse Switch Laser, Coherent, Inc.) generates 100-fs pulses in the near infrared (800 nm). We operated the laser at 10 kHz, used 0.25 s exposure for ablations, and propagated the beam through a home-built 10x Galilean expander.

### Ablation parameters

We ablated samples with 13 to 15-nJ (Yb-fibre), 10-nJ (Ti:Sapphire), and 28-μJ (nanosecond) pulse energies, at 1–10 kHz repetition rate. We used 100 pulses in all cases except for ink ablations with the nanosecond laser, for which we used 5 pulses. We focused pulses with an Olympus UAPO 40×, 1.35 NA oil immersion objective for ink, bundle, and motoneuron ablations and an Olympus 100×, 1.4 NA oil immersion objective for motoneuron ablations. Where possible, laser power and pulse number were set to the minimum setting in which damaged could be observed.

### Measurement of lesion area and estimation of minimal power and beam size

Lesion area at the focal plane was determined by focusing the laser through a coverslip (22 × 40 mm rectangular #1.5 (0.17 mm) thickness) onto a layer of black ink (Sharpie permanent marker, ink side was facing up and away from objective lens). Bright field mages were acquired (acquisition software: MicroManager v.2.0, camera Flash4.0 Hamamatsu, Japan) after lesion at the focal plane, as well as 2 μm above and below. Largest diameter of damage area was quantified from 5 images using ImageJ (FIJI distribution v.1.52).

To estimate the minimum ablation power and damage size, we followed a procedure described by Liu^17^. We set both Ti:Sapphire and Yb-fibre lasers power to the minimum energy in which a damage spot could be observed on a slide covered in black ink frequency was set in both cases at 10 kHz. At least 10 damage spots for each power setting were made on the black slide in each case before increasing power. Power was increased in the smallest possible increments and new damage spots were made to the ink. Power for each setting was measured with an optical power and energy meter (Thorlabs). We plotted the square of the damage diameter against the log of pulse energy.

### Bundle ablation

Bundle ablation was performed in adult animals that express green fluorescent protein (GFP) either pan-neuronally (strain NW1229) or in the amphid neurons (strain NG3416), obtained from *C. elegans* Genetics Center and Gian Garriga, respectively. In all cases, an area of the bundle that included multiple neurites was brought into focus and the laser was aimed at a neurite in the bundle. Images were taken before and after lesion with MicroManager^19^ or Nikon Elements.

### Laser axotomy and regeneration measurements

For laser microsurgery and time-lapse microscopy, *C. elegans* hermaphrodites at the fourth larval stage (L4) were mounted by placing them in a drop of cold, liquid 36% Pluronic F-127 (Sigma-Aldrich) with 1 mM levamisole (Sigma-Aldrich) solution, and pressed between two coverslips^20^. The slides were brought to room temperature, to solidify the Pluronic F-127 gel and immobilize the animals. Laser axotomy was performed using both the Yb-fibre and the nanosecond pulse laser system installed on the same microscope (ASI RAMM open frame with epifluorescence and bright field Olympus optics), for adequate comparison. In both cases, the beam was focused to a diffraction-limited spot that was first located on the live image by lesion of a surface of black ink on a coverslip as described above. The targeted neuron was visually inspected immediately following laser exposure (100–500 ms) to confirm successful axotomy. In some cases, multiple laser exposures were necessary to generate a break in the nerve fibre. Axotomy of D-type motor neurons were done by severing the anterior ventral-dorsal commissures 40–50 μm away from the ventral nerve cord. Neuronal regeneration was assessed 24 hours after axotomy on the same microscope and imaging system.

### Quantification and analysis

Z-stacks were acquired both before and immediately after injury as well as 24 h post injury. Maximum intensity projections were constructed and post injury images (1 min post injury) were analysed to quantify the damage area. Images taken 24 h post injury were analysed to quantify outgrowth. Outgrowth was counted when a new branch extended from the injury site. Image measurements and analysis were carried out with ImageJ software v.1.52, and statistical analysis was done with GraphPad Prism v.8.0.50.

### Statistics and interpretations of results

Most of the D motoneuron regeneration and ectopic outgrowth data is binary: we score whether or not neurons regrow or outgrow. We calculated p-values for these data by Fisher’s exact test. For the size of the injury, we calculated p-values by the unpaired, unequal variance, two-tailed t-test. For the ink damage test, we conducted a one-way analysis of variance (ANOVA). We performed post-hoc comparisons using the Tukey test. Data are represented as average ± standard deviation (SD). * and ** indicate values that differ at p < 0.05 and 0.001 levels, respectively.

The datasets generated during or analysed during the current study are available from the corresponding author on reasonable request.
